# Hepatitis B Virus Diagnosis Using Dried Blood Spots in the D.R. Congo: Overcoming Misdiagnosis to Achieve 2030 WHO Targets

**DOI:** 10.3390/medsci14020271

**Published:** 2026-05-26

**Authors:** Paula Martínez de Aguirre, Silvia Carlos, Samclide Mbikayi, Eduardo Burgueño, David Barquín, Céline Tendobi, Luis Chiva, África Holguín, Gabriel Reina

**Affiliations:** 1 Microbiology Department, Clínica Universidad de Navarra, 31008 Pamplona, Spain; pmartinezdea@unav.es (P.M.d.A.); dbarquin@unav.es (D.B.); 2 Department of Preventive Medicine and Public Health, Universidad de Navarra, 31008 Pamplona, Spain; 3 IdiSNA, Navarra Institute for Health Research, 31008 Pamplona, Spain; 4 Centre Hospitalier Monkole, H7FP+W6P, 10, Monkole Avenue, Kinshasa 4484, Democratic Republic of the Congo; samclide.mbikayi@monkole.cd (S.M.); eburgueno@saludcastillayleon.es (E.B.); baby.tendobi@monkole.cd (C.T.); 5 Department of Gynecology and Obstetrics, Clínica Universidad de Navarra, 28027 Madrid, Spain; lchiva@unav.es; 6 Laboratorio Epidemiología Molecular VIH-1, Hospital Ramón y Cajal-IRYCIS y CIBERESP, Madrid. Ctra. Colmenar Viejo, 28034 Madrid, Spain; africa.holguin@salud.madrid.org

**Keywords:** Hepatitis B, dried blood spot (DBS), sub-Saharan Africa, rapid diagnostic test (RDT), seroprevalence, misdiagnosis

## Abstract

*Background/Objectives*: Hepatitis B remains a major public health concern in the Democratic Republic of the Congo (DRC). This study investigated HBV seroprevalence in Kinshasa and evaluated the diagnostic performance of rapid diagnostic tests (RDTs) compared with dried blood spot (DBS)–based immunoassays. *Methods*: DBS samples collected between 2016 and 2022 were transported to Spain for HBsAg and HBc-Ab testing using two chemiluminescence platforms (ECLIA-COBAS (Roche) and ELFA-miniVIDAS (bioMerieux)). A subset of participants also underwent on-site HBsAg screening using Determine™ (Abbott) RDTs. *Results*: Overall, active HBV infection was detected in 4.3% of participants and resolved infection in 14.3%, with no significant differences by age, sex, cohort, or HIV/HCV status. The RDT showed poor sensitivity (60% (95% CI: 26–88)) but high specificity (100% (95% CI: 98–100)), resulting in a 40% misdiagnosis rate. In contrast, DBS-based HBsAg immunoassays demonstrated excellent diagnostic accuracy, with both platforms achieving 100% sensitivity (ECLIA-COBAS 100%, 95% CI: 66–100; ELFA-miniVIDAS 100%, 95% CI: 99–100) and specificity (ECLIA-COBAS 100%, 95% CI: 98–100; ELFA-miniVIDAS 100%, 95% CI: 99–100). HBc-Ab detection showed platform-dependent variability, with lower sensitivity on ELFA-miniVIDAS (66% (95% CI: 46–82)) compared with ECLIA-COBAS (100% (95% CI: 96–100)). Predictive values were high across all assays, and inter-method agreement for HBsAg between RDT and chemiluminescence was good (Cohen’s kappa 0.71, *p* < 0.001). *Conclusions*: These findings indicate moderate HBV transmission in Kinshasa and highlight the limited reliability of RDT-based screening. DBS proved to be a practical, robust, and scalable sampling method with outstanding diagnostic performance, making it well-suited for HBV testing in low-resource settings.

## 1. Introduction

Hepatitis B virus (HBV) infection is a major public health problem that affects 254 million people worldwide, according to WHO 2022 estimates [1]. The burden is disproportionately high in low- and middle-income countries (LMICs); 63% of newly acquired infections are concentrated in the WHO African Region. In the Democratic Republic of the Congo (DRC), there are 2.8 million total cases and over 100,000 new infections annually [1] and is one of the WHO focus countries.

There are two key aspects to reaching effective HBV management: vaccination of infants and high-risk populations and accurate and accessible diagnosis of active and chronic HBV cases. Immunization in the country has been addressed through the DRC Mashako Plan 2018–2020, which increased vaccination in infants to 70% by 2020. Despite this promising start, outreach to other key populations is defective [2,3], and immunization efforts have been halted by the COVID-19 pandemic and sociopolitical instability. In the DRC, diagnostic coverage is low, and the WHO 2024 report estimates that only 2.4% of infected individuals are aware of their status [1].

Rapid diagnostic tests (RDTs) have been widely employed in resource-limited settings, as they are cost-effective and easy to access. However, they can be affected by transportation and preservation conditions, and result interpretation is often subjective [4]. Other factors such as HBV genotype, the stage of the infection and HIV-HBV coinfection [5] can also negatively impact their performance.

In this context, dried-blood spot samples (DBS) have been proposed as a feasible alternative to traditional blood or serum samples, as they are easily transported and stored and require low blood volume, minimal equipment and basic technical training. First introduced for neonatal screening in the 1950s, they gained increased interest for HIV testing purposes in the 2000s and have since been proven useful in the diagnosis of multiple infectious agents [6,7,8]. Many countries have implemented DBS-based screening programs to improve diagnosis in hard-to-reach populations, and multiple programs in Africa using DBS-based HBV testing have shown promising results [9,10,11,12,13,14], although they are not the standard of practice.

The RDTs primarily used in the DRC target HBsAg, enabling the diagnosis of active cases, but they lack the capacity to detect resolved infections. DBS-based testing strategies, however, can be tested in automated high-throughput platforms multiple times, and by serological and/or molecular methods, which facilitate result confirmation as well as testing scale-up. Some of their drawbacks include the necessity of secondary consultation to communicate results, which can lower retention in care [15], and lesser sensitivity than serum or plasma samples, although studies suggest they still achieve greater sensitivity than RDTs when tested in high-sensitivity platforms [5,14,16,17,18].

The aims of this study are: (1) to describe HBV seroprevalence in Kinshasa (Mont-Ngafula area) in three study groups: individuals at high risk of HIV infection attending Voluntary Counselling and Testing, blood donors from the general population, and women attending a free cervical cancer screening program; (2) to determine the analytic performance of DBS eluates in two chemiluminescence testing platforms in terms of sensitivity, specificity, positive predictive value, and negative predictive value; (3) to compare the performance of the current local HBV RDT-based testing algorithm with the analysis of DBS eluates using a chemiluminescence immunoassay.

## 2. Materials and Methods

### 2.1. Study Design and Population

Between 2016 and 2022, 658 participants were recruited at Centre Hospitalier Monkole (a reference hospital in the Mont-Ngafula area of Kinshasa, DRC) through three different programs (Table 1): (a) the OKAPI (Observation Kinshasa AIDS Prevention Initiative) cohort, which enrolled patients attending a free HIV Voluntary Counselling and Testing (VCT) program (2016–2019); (b) blood donors from the general population (2019–2021); and (c) the ELIKIA project, which included women attending a free cervical cancer screening program (2022).

### 2.2. Sample Collection

Blood samples were collected through venipuncture in an EDTA tube (cohorts 1–2) or by finger prick (cohort 3), and 50–70 µL was spotted on Whatman 903 cards (W-903). Cohorts 1 and 2 were locally screened for HIV using RDT immunoassays (Alere Determine HIV-1/2 Ag/Ab, Abbott, Scarborough, ME, USA). The DBS samples were air-dried (room temperature, 4 h) and stored individually at −20 °C until transported to an external clinical laboratory in Spain. The cold chain was maintained throughout transportation by international courier services and during storage (−80 °C) at facilities in Clinica Universidad de Navarra (Spain). Table 1 summarizes the recruitment and sample collection protocol followed. For this study, patients were re-categorized into three cohorts according to the recruitment program and HIV status.

### 2.3. Serological Assays

#### 2.3.1. Hepatitis B Seroprevalence Study (Spain)

The seroprevalence of HBV was studied at Clínica Universidad de Navarra (Spain) from the venipuncture or finger-pricked blood DBS samples shipped. Elution was manually performed by incubating 2 spots in 1440 µL PBS (BioWhittaker, Lonza, Houston, TX, USA) at 37 °C for 1 h, after which the filter paper residue was discarded, and the sample tested. In some cases, the elution volume was adjusted to account for the low blood sample volume spotted. Finally, remnants were stored at −80 °C.

Two parameters were tested to determine HBV status: HBs antigen (HBs-Ag) and anti-HBcore antibodies (HBc-Ab) by semiquantitative immunoassays performed on either the Elecsys^®^ analyzer (electrochemiluminescence-ECLIA, Roche Diagnostics, Mannheim, Germany) or the miniVIDAS^®^ analyzer (Enzyme-Linked Fluorescent Assay (ELFA), bioMérieux SA, Marcy-l’Etoile, France). All tests were performed following the manufacturer’s protocols. Depending on the DBS eluate results, patients were categorized as “active HBV” (positive HBsAg), “resolved HBV” (negative HBsAg and positive HBc-Ab) or “non-infected” (negative HBsAg and HBc-Ab).

Other parameters such as age and sex were recorded on-site. Other serological assays were carried out at our laboratory in Spain to better characterize the study population. HIV was determined on the same eluate sample by a 4th-generation test (HIV Combi PT Elecsys^®^, Roche Diagnostics, Mannheim, Germany). The HIV-positive population was further characterized: CD4 count, viral load (COBAS AmpliPrep/COBAS Taqman HIV-1, Roche Diagnostics, Mannheim, Germany), and exposure to antiretroviral therapy was recorded. Additionally, the samples were tested for anti-HCV antibodies on the Elecsys^®^ analyzer (Roche Diagnostics, Mannheim, Germany).

Seroprevalence for both active and resolved infections was calculated globally and stratified by sex, age, cohort, HIV and HCV serological status, with their corresponding 95% confidence intervals.

#### 2.3.2. Analytic Performance of HBV Diagnostic Tests

All tests were performed following the manufacturer’s instructions. Prior to the analysis of study samples, both testing platforms were validated using paired plasma/DBS eluates from well-characterized HBV-positive and HBV-negative samples. Elution buffer was also included in this preliminary analysis as a background signal control and to adjust index value interpretation. Diagnostic performance (sensitivity, specificity, positive and negative predictive values) was assessed by comparing each HBsAg or HBc-Ab result with the predefined true-positive reference standard, as described below. Samples were classified as true positives when at least two of the five potential tested parameters were reactive: HBs-Ag ELFA, HBs-Ag ECLIA, HBs-Ag RDT, HBc-Ab ELFA, and HBc-Ab ECLIA. For a subset of samples, molecular testing results were also available.

The suitability of DBS samples for HBV diagnosis (diagnostic performance) was assessed by testing 589 and 584 samples for HBs-Ag and HBc-Ab, respectively, using the ECLIA-COBAS platform. Similarly, 178 and 168 specimens were tested for HBs-Ag and HBc-Ab, respectively, on the ELFA-miniVIDAS platform. Finally, 109 samples were analyzed on both chemiluminescence platforms to determine inter-platform concordance and correlation.

The analytic performance of HBV RDTs was evaluated among blood donors (N = 198, Cohort 2, 2021). Blood samples were obtained through venipuncture and tested in Kinshasa (DRC) using the routine Determine HBs-Ag RDT (Abbott) with plasma/serum specimens. Subsequently, DBS cards were spotted, dried and sent to Clinica Universidad de Navarra (Pamplona, Spain), where the presence of HBsAg and HBc-Ab in DBS eluates was determined, as detailed above.

### 2.4. Statistical Analysis

The results were recorded in Excel and exported to a Stata 15 database (StataCorp, College Station, TX, USA). Descriptive analyses of the participants’ characteristics were conducted. For categorical variables, percentages were compared using Pearson’s Chi test, and for quantitative variables, means were compared using Student’s *t* test.

The diagnostic performance of RDTs and immunoassay-based HBs-Ag and HBc-Ab tests (sensitivity, specificity and positive and negative predictive values) was determined using combined HBs-Ag and HBc-Ab results, respectively, as reference standards.

Concordance and correlation of HBs-Ag and HBc-Ab results between both chemiluminescence platforms were determined using Cohen’s kappa and Spearman’s rho, respectively. All *p*-values < 0.05 were considered statistically significant.

### 2.5. Funding and Ethics

The study was conducted in accordance with the ethical guidelines of the 1975 Declaration of Helsinki. The Ethical Committees of Monkole Hospital/University of Kinshasa (Kinshasa, DRC) and of the University of Navarra (Pamplona, Spain) reviewed and approved the study (40/2015, approved on 19 June 2015; 2017/096, approved on 23 November 2017; and 001/CEFA-MONKOLE/CEL/2018, approved on 22 January 2018). All methods were performed in accordance with relevant guidelines and regulations.

Informed consent was obtained from all subjects involved in the study. All methods were carried out in accordance with relevant guidelines and regulations (CPMP/ICH/135/95). All data and samples were coded and confidentially managed.

This research was funded by the Government of Spain (Fondo de Investigación en Salud-FIS, grant PI16/01908), the Government of Navarre (Grant 045-2015) and Fundación Amigos de Monkole.

## 3. Results

### 3.1. Characteristics of Study Participants

Among the study participants, 251 were recruited through the VCT program, 266 were blood donors and 141 were women attending the free cervical cancer-screening program (CCS). Most participants were female (56.9%), and the overall mean age was 39.8 (SD: 12.6) years (Table 2). A total of 231/658 participants (35.1%) had anti-HIV antibodies, mainly those from Cohort 1.

### 3.2. HBV Seroprevalence in Kinshasa

HBV seroprevalence results are summarized in Table 3. Overall, 28/658 (4.3%) of participants tested positive for HBs-Ag (active HBV) and 14.3% tested positive for HBc-Ab and negative HBs-Ag (resolved HBV). No significant differences were found between age or sex groups, nor across cohorts. Similar seroprevalence was found in HIV-positive and HIV-negative populations. Although not statistically significant, anti-HCV-positive patients appeared to have a higher rate of active HBV infection (11.8% vs. 4.2%).

### 3.3. HBV Infection Among HIV-High Risk and HIV+ Population in Kinshasa

HIV-positive patients with active HBV infection (5.2%) tended to have lower CD4 counts and higher HIV viral loads than those with resolved infection, although these differences were not statistically significant (Table 4).

Most participants with an active HBV infection reported being on ART (9/12, 75%). This association was not found in patients with resolved infections (12/25) or those not infected with HBV.

HIV subtype was studied in HIV-positive patients, and strains from 8/12 active HBV-HIV coinfected patients and strains from 18/29 resolved HBV-HIV patients were subtyped. HIV-1 subtype A was the most prevalent (3/8) variant among active infections. By contrast, among resolved HBV cases, subtype A was less prevalent, while other strains were more frequently found.

### 3.4. Analytic Performance of HBV Diagnostic Tests: RDT vs. ECLIA and ELFA

Among the 198 samples tested for HBsAg using both RDT and immunoassay platforms, 10 were considered true positives (Table 5). The HBV RDT local test had a positive result for 6/10 samples, reaching a 60% (95% CI: 26–88%) sensitivity and 100% (95% CI:98–100%) specificity. The inter-rate concordance between RDT and ECLIA-COBAS platform was good (Cohen’s kappa 0.71, *p* < 0.001).

HBsAg immunoassays identified all true-positive cases and demonstrated excellent sensitivity and specificity (Table 5). In contrast, HBc-Ab detection showed greater variability between platforms: the ELFA-miniVIDAS assay missed 10 of 29 positive samples, resulting in markedly lower sensitivity (65.5%, 95% CI: 46–82) compared with the ECLIA-COBAS platform, which achieved 100% sensitivity (95% CI: 96–100). Despite these differences, both platforms showed satisfactory positive and negative predictive values.

### 3.5. Analytic Performance of HBV Diagnostic Tests: Immuno-Chemiluminescence Platforms

A total of 109 DBS samples from Cohort 2 were tested in parallel on both semi-automated platforms for HBsAg, and 94 samples were similarly evaluated for HBc-Ab. The ECLIA-COBAS platform identified one false-positive HBsAg result and detected ten HBc-Ab-positive cases that were missed by the ELFA-miniVIDAS assay.

**Figure 1:**
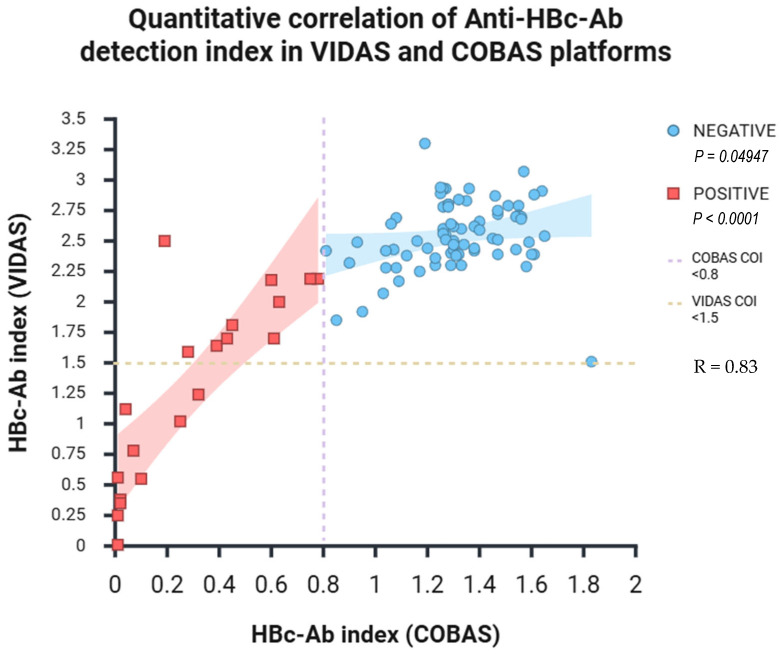
Correlation of HBc-Ab antibodies detection index in parallel-tested DBS eluates (n = 109); COI: cut-off index; HBc-Ab VIDAS-COI (<1.5), HBc-Ab COBAS-COI (<0.82).

Cohen’s kappa showed almost perfect agreement in HBs-Ag determination (k = 0.89, 95% CI= 0.64–1.00, *p* < 0.001) and moderate agreement in the case of HBc-Ab (k = 0.57, 95% CI = 0.3–0.85, *p* < 0.001). Quantitative correlation was also good for the HBc-Ab index (Spearman’s rho = 0.61, *p* < 0.001) (Figure 1). HBsAg index correlation was not calculated due to the limited number of positive samples.

## 4. Discussion

This study offers a comprehensive overview of HBV seroprevalence in the Mont-Ngafula area of Kinshasa (DRC) across three distinct population groups: individuals at high risk of HIV infection, blood donors from the general population, and middle-aged women attending a cervical cancer screening program. Overall, active HBV infection was detected in 4.3% of participants, while 14.3% showed markers of resolved infection. Among HIV-positive individuals, active and resolved HBV infections were identified in 5.2% and 11.7%, respectively. In the smaller subgroup with HCV co-infection, active and resolved HBV infection rates were 11.8% and 13.3%, respectively.

Studies in other sub-Saharan African countries indicate a heterogeneous distribution of HBV, with high seroprevalence of active infection in Mali (14.8%) [19], Ghana (13%) [20], Cameroon (11.2%) [21], Benin (10%) [22], the Republic of Congo (6.6–9.9%) [23] and Nigeria (7%) [22,24], and a similar 4% in Ethiopia [25]. Our study found a 4.1% seroprevalence, in agreement with other studies in the DRC that indicate intermediate to high seroprevalence by WHO criteria [3]. In regions like Lubumbashi, between 2.7% and 9.3% [26,27,28,29] active HBV has been reported, but data from the Kinshasa area shows higher agreement, with a seroprevalence between 4 and 5.9% [30,31,32]. A systematic review by Schweitzer et al. reported 5.9% [33] active HBV infections (1965–2013) and more recently Shindano et al. estimated a 4.9% [31] seroprevalence (2000–2016) in DRC. The declining seroprevalence observed could indicate that vaccination efforts are starting to have an effect.

No significant differences by sex, age, or study cohort were observed in our analysis. Although some studies suggest potential age- and sex-related variations—with higher HBV prevalence reported among men and individuals aged 18–45 years [31]—our findings did not reflect these patterns. DBS-based data from the 2013–2014 Demographic and Health Survey in the DRC [34] also showed higher seroprevalence in adults than in children, suggesting both horizontal and vertical transmission. However, that survey differed substantially from our study population, as only 0.9% of participants were older than 41 years and no HIV–HBV coinfections were identified. Additionally, studies in pregnant women report active HBV infection rates of 3.9% [4,35] and up to 4.7% among those living with HIV [36], values comparable to the 4.4% observed among women in our cohort.

One of the recruitment settings was the blood bank, which in countries like the DRC relies predominantly on family replacement donors (typically young male individuals who donate to help a particular relative or friend). The seroprevalence observed in this group did not differ significantly from that in the other cohorts. Previous studies suggest that intrafamilial transmission (both vertical and horizontal) has a predominant role in the country [37,38], as also observed in other sub-Saharan settings [39], which could explain the absence of significant differences in seroprevalence between cohorts.

Our results show that the routine RDT-based testing algorithm on site has a 40% misdiagnosis rate when compared against DBS samples tested on immunoassays, which makes it deficient as a screening test. At the time the samples were obtained, blood-borne virus screening in the blood bank was performed using RDTs, but protocols have since been updated to comply with national health recommendations. Reviews and meta-analyses assessing RDT, EIA and PCR testing methods similarly reported high variation in accuracy, unrelated to sample type, with 12 RDT tests reporting sensitivity equal to or under 60% [4,5]. Sensitivity was reduced when molecular methods were used as reference standard in patients with HIV-HBV coinfection and when studies were performed in the field compared to laboratory settings. In the DRC, the most frequently described HIV subtypes are A, E and D [26,40]; both E and D have been associated with reduced RDT sensitivity, which could partly account for poor RDT performance.

Viral load was higher and CD4 counts were lower in patients with an active HBV infection, although no statistically significant differences were found among active and resolved infections, nor between infected and non-infected seropositive patients, as described in other studies [20].

Despite HIV, HBV and HCV sharing transmission routes, the diagnosis remains problematic in many sub-Saharan populations and is not integrated into successful HIV screening programs such as Voluntary Counselling and Testing. Furthermore, HBV testing often relies on RDTs, which have been found to be negatively impacted by HIV-HBV coinfection and lamivudine-containing ART regimens [5]. The prevalence of HIV-active HBV coinfection ranges from 1.1% [10] to 8.4% [20], depending on the country and study population. Data from the DRC is scarce and heterogeneous. The 2023 UNAIDS datasheet reports HCV-HIV coinfection but not HBV [41]. Studies in pregnant women have reported coinfection rates ranging from 3.3% [35,36] to 7.9% [26,42], and in blood donors, 1% coinfection was reported in 2001 [43].

DBS use has been thoroughly described for molecular [8,16,44,45] and serological [11,12,13,14,15,44] detection of blood-borne viruses, including HBV, showing excellent correlation with serum and/or plasma and good stability even at room temperature. Despite these promising reports, and the inclusion of DBS in WHO viral hepatitis screening guidelines [3], most manufacturers are yet to consider DBS eluates as a viable sample.

Our results support the good performance of DBS eluates for HBV screening by immunoassay at the population level. They were easily obtained and preserved during transportation. After conservation and elution, they showed outstanding testing accuracy by immunoassay, and DBS-based testing showed superiority over RDT-based screening algorithms. The comparison between the ELFA (miniVIDAS) and ECLIA (Roche) platforms showed almost perfect agreement in testing HBs-Ag using DBS eluates, which is the preferred testing method for HBV infection screening. The qualitative detection of HBc-Ab had moderate agreement between these two platforms, with good correlation in their index values.

The main strength of this HBV seroprevalence study is the robustness of the testing methods. After validation of DBS samples as accurate, seroprevalence was determined by immunoassay on two platforms: ELFA and ELISA. Additionally, the use of DBS allowed testing of HBc antibodies, which is not commonly reported in seroprevalence studies and is necessary to detect chronic and resolved infections. Although geographically limited, samples were obtained from three recruitment programs with very different characteristics, adding diversity to the study population. HIV-positive individuals remain a key population given their higher risk of HBV infection and HBV-related complications.

This study has several limitations. First, the study populations were restricted to the Kinshasa area, and the findings may not fully reflect the epidemiological situation across the DRC. However, previous studies indicate that blood donors in the DRC are broadly representative of the general population in terms of age distribution and overall health status [46]. Second, testing capacity was constrained by the limited volume of DBS eluates, preventing all samples from being assessed across every diagnostic platform. Additionally, some sociodemographic data were unavailable for a subset of participants. Finally, the absence of a formal gold standard (paired plasma samples) limited the ability to directly compare assay performance. However, this limitation was mitigated by defining true-positive cases through the combined interpretation of all available diagnostic markers, thereby approximating real-world clinical practice, where complementary serological indicators are routinely integrated for HBV diagnosis.

## 5. Conclusions

HBV seroprevalence in Kinshasa reflects a pattern of moderate viral transmission. Our findings demonstrate that reliance on rapid diagnostic tests as the primary screening tool leads to approximately 40% misdiagnosis, substantially limiting the effectiveness of current testing algorithms. In contrast, DBS-based approaches—alone or combined with other diagnostic technologies—offer higher accuracy, improve data quality, and enhance population outreach. These results indicate that the challenge of hepatitis B diagnosis remains insufficiently addressed in low-resource settings, where RDT-based practices continue to lack adequate sensitivity and specificity. Moreover, this study demonstrates that DBS specimens are practical, reliable, and well-suited for real-world low-resource environments, supporting their use in large-scale HBV serosurveys and routine surveillance programs.

## Figures and Tables

**Table 1:** Study population and sample collection characteristics (N = 658).

Study Cohort	Recruitment Program		Sample Collection		On-Site RDT
Cohort 1	HIV Voluntary Counselling and Testing	2016–2019 N = 251	Venipuncture whole blood	5 spots, W-903 card	RDT HIV+
Cohort 2	Blood donors	2019–2021 N = 266	Venipuncture whole blood	5 spots W-903 card	RDT HIV− RDT HBV ^1^
Cohort 3	ELIKIA	2022 N = 141	Finger-pricked	2–3 spots W-903 card	**-**

RDT: Rapid Diagnostic Test; ^1^ On-site HBV RDT testing was performed on 207 patients in 2021.

**Table 2:** Sociodemographic and clinical characteristics of the participants across study cohorts.

	Cohort 1 n = 251	Cohort 2 n = 266	Cohort 3 n = 141	TOTAL
Recruitment program	VCT	BD	CCS	658
Sex ^1^ (female) (%)	64	18.7	100	56.9
Age (years) (mean, SD)	42.8 (13.0)	34.5 (10.9)	45.1 (11.0)	39.8 (12.6)
HIV positive (%)	88.5	0	6.4	35.1
HCV positive ^2^ (%)	5.5	2.2	0.7	3.0

VCT: Voluntary Counselling and Testing; BD: Blood Donors; CCS: Cervical Cancer Screening (N = 141). ^1^ Sex was not known for 40/251 patients in Cohort 1 and 68/266 in Cohort 2. ^2^ HCV status was determined through anti-HCV-antibody testing on DBS.

**Table 3:** Seroprevalence of active/resolved HBV infections by cohort, sex, age, and HIV, HCV status.

			Active HBV (%)	Resolved HBV (%)	Total (%)
Study population	Cohort 1	n = 251	4.8	11.6	16.3
	Cohort 2	n = 266	4.5	16.9	21.4
	Cohort 3	n = 141	2.8	11.4	14.2
Sex	Male	n = 237	4.6	16.0	20.7
	Female	n = 313	4.5	11.2	15.7
Age	<40 years	n = 318	3.8	12.6	16.4
	>40 years	n = 297	5.4	15.5	20.9
HIV	Positive	n = 231	5.2	11.7	16.9
	Negative	n = 427	3.8	14.8	18.5
HCV *	Positive	n = 17	11.8	13.3	23.5
	Negative	n = 550	4.2	12.4	16.6
	TOTAL		4.3	14.3	17.9

Active HBV infection: (+) HBs-Ag; Resolved HBV infection: (+) HBc-Ab and (−) HBs-Ag. * HCV status was determined through anti-HCV-antibody testing on DBS.

**Table 4:** HBV seroprevalence within Cohort 1 (HIV-high risk and HIV+).

			HBV Infection					
			Active (n = 12)		Resolved (n = 29)		Non-Infected (n = 210)	
CD4 count (cells/µL), mean (SD)			188.6	(81.6)	280.2	(269.7)	275.3	(181.8)
HIV viral load (log), mean (SD)			3.5	(1.1)	3.0	(1.9)	3.2	(1.7)
			(%)		(%)		(%)	
HIV-1 subtype	A		37.5		5.6		19.0	
	Other subtypes		-		27.8		23.2	
	CRF		12.5		5.6		11.3	
	URF/unclassified		50		61.1		46.5	
ART regimen	Naïve		16.7		48.3		39.1	
	Experienced		75.0		41.4		49.1	
	(of which)	TDF/FTC	33.3		50.0		52.4	
		3TC	100		91.7		98.1	
	Unknown		8.3		10.3		11.9	
TOTAL (%)			4.8		11.6		83.7	

Active HBV infection: (+) HBs-Ag; Resolved HBV infection: (+) HBc-Ab and (−) HBs-Ag; Non-infected: (−) HBc-Ab and (−) HBs-Ag; CRF: circulating recombinant forms; URF: unique recombinant forms. ART: antiretroviral therapy; TDF/FTC: tenofovir/emtricitabine; 3TC: lamivudine.

**Table 5:** Analytical performance of HBV rapid diagnostic tests and dried blood spot–based HBV immunodiagnosis (95% CI).

	HBs-Ag			HBc-Ab	
	RDT (n = 198)	miniVIDAS (n = 178)	COBAS (n = 589)	miniVIDAS (n = 168)	COBAS (n = 584)
True positive	6/10	9/9	24/24	19/29	103/103
True negative	188/188	169/169	563/565	139/139	481/481
SENSITIVITY (%)	60.0 (26–88)	100 (66–100)	100 (86–100)	65.5 (46–82)	100 (96–100)
SPECIFICITY (%)	100 (98–100)	100 (98–100)	99.6 (99–100)	100 (97–100)	100 (99–100)
PPV (%)	100 (54–100)	100 (66–100)	92.3 (75–99)	100 (82–100)	100 (96–100)
NPV (%)	97.9 (95–99)	100 (98–100)	100 (99–100)	93.3 (88–97)	100 (99–100)

RDT: Rapid Diagnostic Test; PPV: Positive Predictive Value; NPV: Negative Predictive Value.

## Data Availability

The data presented in this study are openly available in Harvard Dataverse at https://dataverse.harvard.edu/citation?persistentId=doi:10.7910/DVN/SASZSV (accessed on 13 April 2026).

## Abbreviations

The following abbreviations are used in this manuscript:

HBV	Hepatitis B Virus
WHO	World Health Organization
LMICs	Low- and Middle-Income Countries
DRC	Democratic Republic of the Congo
RDTs	Rapid Diagnostic Tests
HIV	Human Immunodeficiency Virus
DBS	Dried Blood Spot
OKAPI	Observational Kinshasa AIDS Prevention Initiative
VCT	Voluntary Counselling and Testing
HCV	Hepatitis C Virus
HBs-Ag	Hepatitis B Surface Antigen
HBc-Ab	Hepatitis B Core Antibodies
BD	Blood donors
CCS	Cervical Cancer Screening
ART	Anti-Retroviral Therapy
